# Metatranscriptomic and metagenomic analyses of cotton aphids (*Aphis gossypii*) collected from cotton fields in Alabama, USA

**DOI:** 10.3389/finsc.2025.1461588

**Published:** 2025-02-26

**Authors:** Chaoyang Zhao, Cesar Escalante, Alana L. Jacobson, Kipling S. Balkcom, Kassie N. Conner, Kathleen M. Martin

**Affiliations:** ^1^ National Soil Dynamics Laboratory, The United States Department of Agriculture - Agricultural Research Service (USDA-ARS), Auburn, AL, United States; ^2^ Department of Entomology and Plant Pathology, Auburn University, Auburn, AL, United States; ^3^ Alabama Cooperative Extension System, Auburn University, Auburn, AL, United States

**Keywords:** melon aphid, RNA sequencing, CLRDV, microbiome, symbiont, taxonomy, polyphagy

## Introduction

Aphids are among the most destructive insect pests to crops. Based on the degree of their host specialization, aphids, like other herbivorous insects, have been grouped into three categories: monophagous, oligophagous, and polyphagous (1). Monophagous aphids feed on only one or a few closely related plant species, often of a single genus, oligophagous aphids feed on several plant species of the same family, and polyphagous aphids feed on plants that belong to more than one family. Polyphagous aphids are considered generalist herbivores, comprising less than half of the total aphid species (2, 3). However, this polyphagous nature allows generalist aphids to disseminate plant pathogens to a wide range of host plants (3, 4).

The cotton aphid (or melon aphid), *Aphis gossypii* Glover, is a highly polyphagous aphid species that can feed on at least 700 plant species in numerous families including Asteraceae, Cucurbitaceae, Malvaceae, Rutaceae, Solanaceae, and Fabaceae (5–7). Population studies of *A. gossypii* have shown that diversity is mainly associated with differences in host plant preference. Moreover, several plant host-specialized biotypes have been documented (8–10). Other factors including geography, climate, and pesticide use can also contribute to shaping its population structure (11, 12). Interestingly, profiles of microbial symbionts, on which aphids are dependent in numerous physiological processes, may vary in different *A. gossypii* biotypes and populations, suggesting specialized interactions evolved between *A. gossypii* and its microbial symbionts under selection pressure exerted by a variety of environmental factors (13–15). Hence, a population-specific microbiome analysis is crucial to understanding aphid-microbe interactions in locally adapted *A. gossypii*.

As a worldwide distributed agricultural pest, *A. gossypii* is responsible for severe yield losses of many economically important crops such as cotton, cucumber, and citrus (5, 7). Besides injuring plants directly by sucking the sap, while feeding, it secretes honeydew which fosters growth of sooty mold that can block sunlight and decrease photosynthesis processes within the plant (16). Moreover, *A. gossypii* is important for its ability to transmit over 75 plant viruses (17) and was ranked the second most competent aphid species in terms of number of potyviruses it vectors (3). In cotton, *A. gossypii* transmits several viruses including cotton leaf roll dwarf virus (CLRDV), cotton anthocyanosis virus, and cotton bunchy top virus, posing a severe threat to cotton production (18–20).

In the Southeast USA, cotton is one of the most economically important crops, and *A. gossypii* is a major insect pest of cotton and the only known vector of CLRDV. As a primary cotton-growing region in the Southeast, Alabama also reported the first occurrence of CLRDV in 2017 (21). This virus was later detected throughout the Southeast (22, 23). Given the significant economic impact of *A. gossypii* in Alabama, we performed metatranscriptomic and metagenomic analyses on locally collected cotton aphids to decipher their microbiota.

## Value of the data

These sequencing datasets provide genetic information, at both RNA and DNA levels, of symbiont microbes and their overall community composition in a local *A. gossypii* population from Alabama, USA. The microbiome data can be used to identify *A. gossypii*-associated and transmitted plant pathogens and discover insect-infecting microbes for aphid biocontrol. In addition, plant species identified in the sequencing data from the whole aphid, will provide insights into the plant host range of *A. gossypii* in the locality tested.

## Materials and methods

### Aphid collection and nucleic acid extraction

Alate *A. gossypii* were collected from the upper 1/3 of the canopy on different cotton plants in the cotton fields of South Alabama when cotton was flowering. Samples were stored in RNAlater™ Stabilization Solution (Thermo Fisher Scientific Inc., Waltham, MA, USA) at -80°C. Five alataes collected on 6/28/2020 in Brewton, AL (31.143390, -87.049873) and another five collected on 6/28/2021 in South Newville, AL (31.389972, -85.421248) were pooled for total RNA and DNA extraction using the Quick-DNA/RNA™ MiniPrep Plus kit (Zymo Research, Irvine, CA, USA). DNA was digested from total RNA using the on-column method described by the RNA Clean & Concentrator-5 kit (Zymo Research, Irvine, CA, USA). Nucleic acid concentration and quality were assessed in a spectrophotometer (NanoDrop^®^) and an automated electrophoresis system (Tape Station 4200, Agilent Technologies, Santa Clara, CA, USA), respectively.

### Sample sequencing

Library preparation and Illumina sequencing were conducted at Novogene Corp. Inc. (Sacramento, CA, USA).

For RNA sequencing, ribosomal RNAs from both eukaryotes and prokaryotes were first depleted from total RNA samples using the Ribo-Zero rRNA removal kit (Illumina, USA). The remaining RNAs were fragmented into ~250 to 300 bp and then reverse-transcribed into double-stranded cDNAs. For metagenomic sequencing, 1 µg of genomic DNA was randomly sheared into short fragments of approximately 350 bp. The double-stranded cDNAs (for RNA sequencing) and sheared genomic DNA fragments (for DNA sequencing) were subsequently end repaired to produce blunt ends, added with a single ‘A’ nucleotide at the 3’ ends, and further ligated with Illumina adapters. After fragment size selection and PCR amplification, the prepared metatranscriptomic and metagenomic libraries were sequenced on the Illumina NovaSeq platform (Illumina, CA, USA) with pair-end 150 mode.

### Assembly of metatranscriptome and metagenome

To generate a metatranscriptome, RNA raw reads were first preprocessed by trimming adaptors and removing low-quality reads using Trimmomatic (v0.39) in paired end mode (24). Parameters for Illumina clip were seed mismatches = 2, palindrome clip threshold = 30, and simple clip threshold = 10. Other parameters included the sliding window trimming with a window size = 5, required quality = 20, and minimum read length = 50. Clean reads were then aligned to the *A. gossypii* genome (NCBI accession GCF_020184175.1) (25) using the BWA-MEM mapping tool (v0.7.17) with its default parameters (26). Unmapped paired reads were assembled to create metatranscriptomic contigs using SPAdes (v3.15.5) in meta mode (27).

A metagenomic assembly was similarly generated following these three steps: 1) preprocessing of DNA raw reads, 2) mapping of clean reads to the reference genome, and 3) assembling of unmapped paired reads. For Step 1, Readfq (v8; https://github.com/cjfields/readfq) was used to trim adaptors and remove the low-quality reads that have: a) more than 40 low-quality bases with Q-value < 38, b) more than 10 ambiguous nucleotides “N”, or c) more than 15 bp’s overlap with adaptors. Step 2 was conducted using BWA-MEM as described above. For Step 3, Megahit (v1.2.9) was used at the default setting to generate metagenomic contigs (28).

### Taxonomic analysis

Contigs longer than 400 bp were retrieved for taxonomic analysis. Contig sequences were first aligned to a preformatted NCBI non-redundant (NR) reference database downloaded on August 28, 2023, with the BLASTX function by running DIAMOND (v2.1.8) (29). The output was written in DAA (DIAMOND alignment archive) format, which was then used for Meganization, an approach of performing taxonomic and functional binning of the sequences (30). The DAA file was run against the MEGAN database ‘megan-map-Feb2022.db’ in long read mode, using MEGANIZER, a program included in the MEGAN package (v6_25_3) (31). Lastly, a taxonomic analysis was conducted using MEGAN, in interactive mode, to determine kingdom and genus level assignations for all contigs.

## Data description

### Sample sequencing and read assembly

Total RNA and DNA extracted from the *A. gossypii* sample, consisting of 10 field-collected alataes, had high purity (OD260/280 > 2.0) and high quality (RIN = 8.3). A total of 88,776,140 and 84,900,570 raw reads were obtained from the Illumina sequencing of RNA (AAL8R) and DNA (AAL8D) samples, respectively, which, after preprocessing to remove adaptors and low-quality reads, yielded 86,527,106 and 84,867,588 clean reads (**Supplementary Table S1**). The GC content of DNA reads was lower (26.77%) than the RNA reads (39.00%) but similar to PacBio reads (27.26-27.99%) of the published *A. gossypii* genome used as reference (25). Mapping of RNA and DNA reads to the *A. gossypii* genome revealed 49.21% and 98.17% of genome coverage, respectively. A total of 17,914,277 and 5,563,338 potentially non-host RNA and DNA reads were unmapped, accounting for 20.70% and 6.56% of their total clean read numbers (**Supplementary Table S1**).

Two *de novo* assemblies were generated: one from the AAL8R and the other from the AAL8D non-host reads not mapped to the *A. gossypii* genome. The AAL8R assembly consisted of 23,101 contigs with an average length of 365 bp and a medium (N50) length of 337 bp. The AAL8D assembly consisted of 11,415 contigs with an average length of 984 bp and a medium length of 1,390 bp (**Supplementary Table S1**). Contigs longer than 400 bp, including 3,804 AAL8R contigs and 8,454 AAL8D contigs, were finally selected for taxonomic annotation.

### Taxonomic annotation

Taxonomic analysis of the non-host reads using Kraken2 (32) identified 1,309,526 (14.62%) unclassified RNA reads and 668,479 (24.03%) unclassified DNA reads. Bacterial reads were the most abundant in both libraries, comprising 7,230,419 (80.73%) of RNA reads and 1,673,778 (60.17%) of DNA reads. Other taxa accounting for ≥ 1% of the non-host reads included viruses (294,166 reads, 3.28%) and Eukaryota (96,840 reads, 1.08%) in AAL8R, and Eukaryota (393937 reads, 14.17%) in AAL8D.

**Figure 1:**
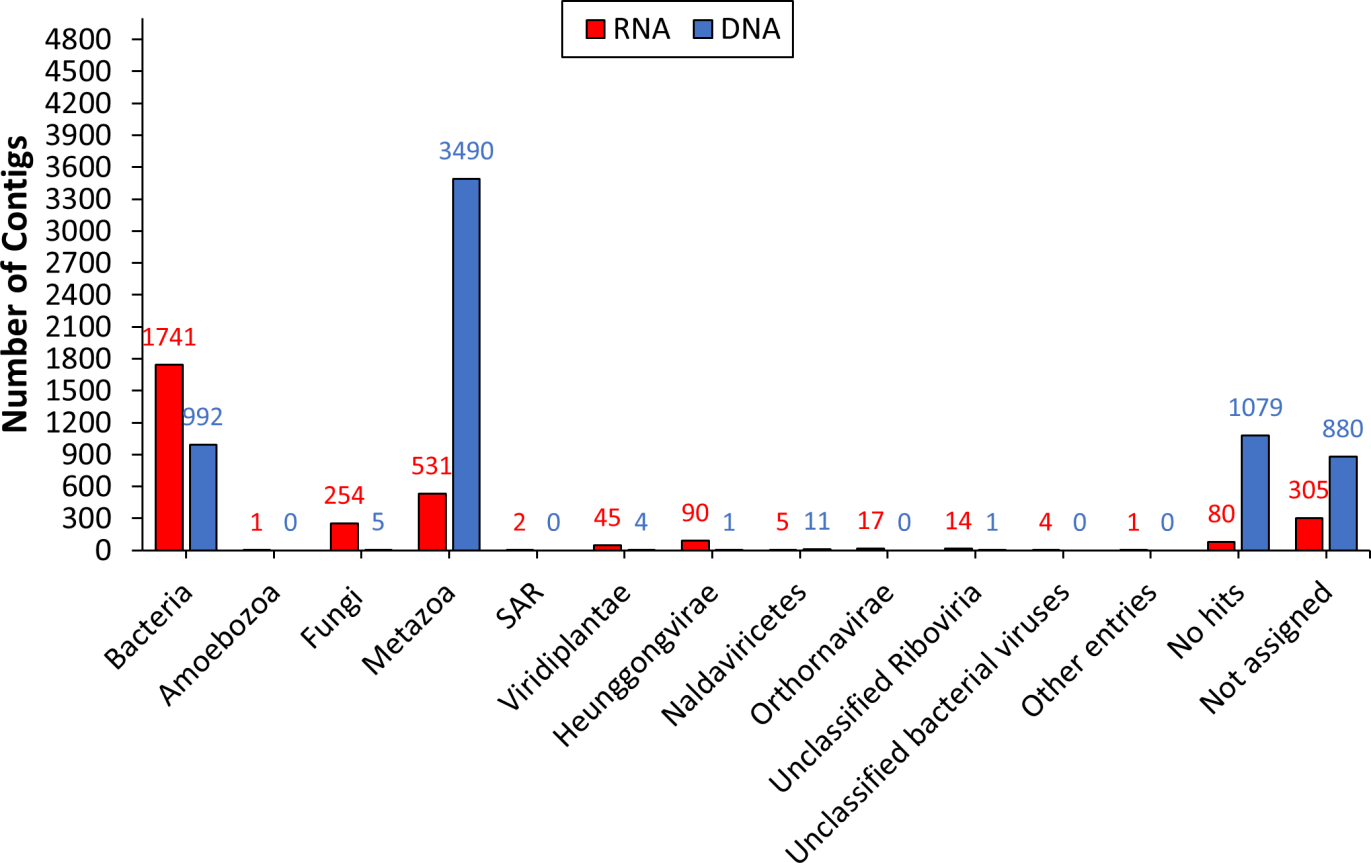
Taxonomic annotation of the RNA (AAL8R) and DNA (AAL8D) contigs at the kingdom level.

The acquired metatranscriptomic and metagenomic contigs were annotated at the kingdom and genus levels using the DIAMOND+MEGAN taxonomic analysis approach (30). Over half of the contigs in both RNA and DNA datasets assembled from non-host reads were classified into specific kingdoms. This included 2704 (71%) AAL8R and 4504 (53%) AAL8D contigs (**Figure 1**). “Bacteria”, “Metazoa”, and “Fungi” were the three most abundant kingdoms for AAL8R. “Metazoa”, “Bacteria”, and “Naldaviricetes” were most abundant for AAL8D. In both the RNA and DNA datasets, a high proportion of sequences received a “Metazoa” assignation. This is likely the result of reads that did not map to the reference genome due to the presence of sequence gaps and as a result were designated as non-host reads (25). Genetic variation between the reference genome, obtained with aphids collected in China (25), and those used in this experiment, collected in Alabama, may be another factor that led to the designation of some reads as non-host. Contigs assembled from these “non-host” reads consequently received the “Metazoa” assignation.

**Figure 2:**
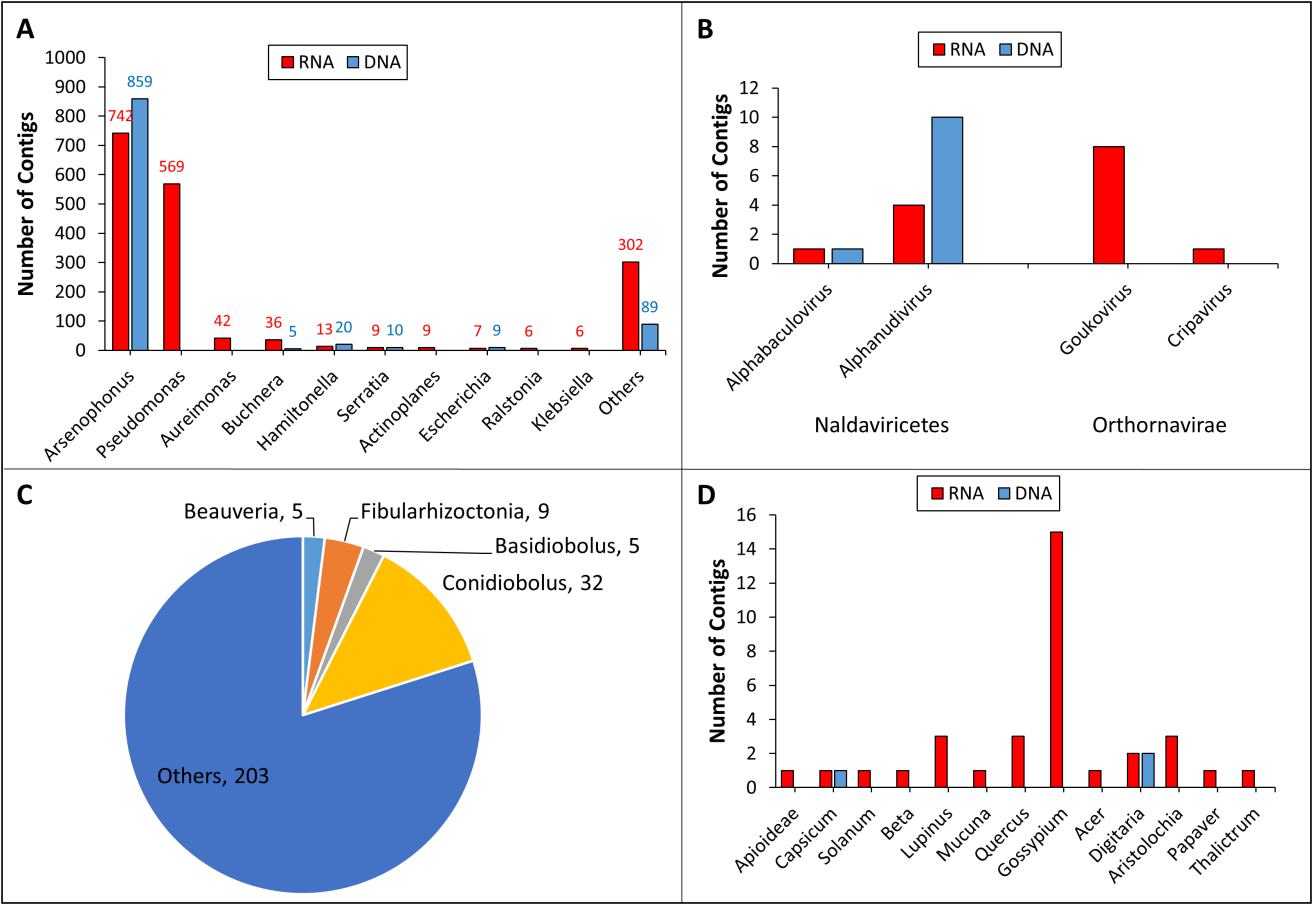
Taxonomic annotation of the RNA (AAL8R) and DNA (AAL8D) contigs at the genus level. The number of contigs assigned to the genera of bacteria **(A)**, Naldaviricetes (**B**, left), Orthornavirae (**B**, right), fungi **(C)**, and plants **(D)** are shown. For bacteria and fungi, only genera containing ≥ 5 contigs are listed. No fungal genera in AAL8D contain ≥ 5 contigs. Fungal genera and their contig numbers in AAL8R are indicated in the pie chart **(C)**.

Previous studies showed that the microbiome of *A. gossypii* can be determined by a variety of factors, including plant host, geography, and life stage (13–15, 33, 34). Our genus-level taxonomic analysis on bacterial contigs indicated that the genus *Arsenophonus* was the most dominant group of symbionts in both AAL8R and AAL8D samples (**Figure 2A**). *Arsenophonus* species are known as male-killing facultative symbionts found in a broad range of arthropod hosts (35, 36). Aside from acting as son killers to benefit female offspring (37), some *Arsenophonus* species were recognized as insect-vectored plant pathogens (38). In aphids, members of *Arsenophonus* can also play a role in parasitoid defense (39) and plant host specification (40).

Our analysis demonstrated that *Pseudomonas* was the second most dominant bacterial genus (569 contigs) in the AAL8R sample (**Figure 2A**). Like *Arsenophonus*, *Pseudomonas* has been shown to interact with its insect host in a multifaceted manner: while some species are entomopathogenic, others may be beneficial endosymbionts of insects or insect-vectored plant pathogens (41). Other bacterial genera with ≥ 10 contigs in either AAL8R or AAL8D included *Aureimonas*, *Buchnera*, *Hamiltonella*, and *Serratia* (**Figure 2A**). Among these, *Aureimonas* was found in a recent cotton microbiome study (42) but has not been reported as an aphid symbiont. Given that cotton components were likely present in the gut of *A. gossypii* collected in the cotton field, it is not possible to discriminate whether *Aureimonas* DNA reads originated from the aphid or the cotton host. By contrast, *Buchnera* is a well-studied primary endosymbiont present in almost all aphid species (43). Despite the contig numbers not being the highest, Kraken2 taxonomic analysis indicated that the number of reads assigned to *Buchnera* comprised 78.79% (7,056,145 reads) and 56.7% (1,577,306 reads) of the non-host RNA and DNA reads, respectively. This suggests that using contig numbers to infer the abundance of a taxon could be inaccurate, as it does not take into account many factors, such as genome size, contig length, and sequencing depth. However, the number of contigs represents a useful metric for initial assessments, providing a general overview of the taxonomic composition within a sample, especially when combined with other analytical methods. Previous studies using 16S rRNA sequencing have confirmed the presence of several bacterial genera in *A. gossypii*, including *Buchnera*, *Arsenophonus*, *Pseudomonas*, *Hamiltonella*, and *Serratia* (13–15). While *Hamiltonella* was shown to mainly play a role in stress tolerance and parasitoid defense in insects, *Serratia* has been shown to be symbiotic or pathogenic to its insect host (44–46).

Two genera of DNA viruses, *Alphabaculovirus* and *Aplhanudivirus*, were detected in both RNA and DNA samples (**Figure 2B**). Detection of DNA viruses in the RNA sample suggested that they were actively replicating in the host cells. *Alphabaculovirus* and *Aplhanudivirus* are double-stranded DNA (dsDNA) viruses that infect insects (47, 48). Therefore, we suspect that the contigs of these two genera in the samples could correspond to DNA viruses of *A. gossypii*. Further analysis is needed to determine their biological and molecular properties. We also found sequences of two genera of putative RNA viruses, *Goukovirus* and *Cripavirus* (**Figure 2B**), whose members are known to infect insects (49, 50). Although the aphid-transmitted cotton virus, CLRDV, is widely distributed in cotton fields (23), we did not find CLRDV contigs in this study, possibly due to the relatively small aphid sampling size.

Taxonomic analysis on the fungal contigs revealed that *Conidiobolus*, *Fibularhizoctonia*, *Basidiobolus*, and *Beauveria* were the most abundant genera with at least 5 contigs each (**Figure 2C**). Notably, *Conidiobolus* and *Beauveria* encompass significant entomopathogenic species like *B. bassiana*, which has been employed as a biological insecticide to manage a diverse array of insect pests (51, 52). Despite the prevalence of *Neozygites fresenii*, a naturally occurring fungal pathogen of *A. gossypii* in the Southeast USA (53, 54), we did not identify any contigs assigned to the *Neozygites* genus. This may be the result of the small aphid sample size used in this study.

Our analysis, mostly from the RNA sample, showed that the highest number of plant contigs were assigned to the genus *Gossypium*. This is not surprising as the aphids were collected in cotton fields. However, we also detected at least one contig for twelve other plant genera belonging to several families (**Figure 2D**). Although these spurious hits in the database are not conclusive, they might represent remains of plant hosts fed upon by the aphids. Should this be true, this finding supports the well-established fact that *A. gossypii* is polyphagous but also suggests that the aphids we collected moved in and out of the cotton field and had fed on a variety of plants.

In conclusion, through sequencing *A. gossypii* alataes collected in Alabama, USA, we generated two *de novo* assemblies: a metatranscriptome and a metagenome. The DIAMOND+MEGAN taxonomic analyses on these assemblies uncovered putative sequences of a variety of organisms that may form complex interaction networks associated with *A. gossypii*. These protocols can be applied not only for microbiome analysis but also to investigate the host range of herbivorous insect species. Additionally, the DNA reads can be used for population genomics research, the RNA reads can be used to enhance gene annotation, and both RNA and DNA reads can contribute to refining the assembly of the *A. gossypii* genome.

## Data Availability

The datasets presented in this study can be found in online repositories. The names of the repository/repositories and accession number(s) can be found below: https://www.ncbi.nlm.nih.gov/genbank/, PRJNA1113681 https://www.ncbi.nlm.nih.gov/genbank/, SAMN41462905 https://www.ncbi.nlm.nih.gov/genbank/, SAMN41462906.
